# Combined Proteotranscriptomic-Based Strategy to Discover Novel Antimicrobial Peptides from Cone Snails

**DOI:** 10.3390/biomedicines9040344

**Published:** 2021-03-29

**Authors:** Anicet Ebou, Dominique Koua, Audrey Addablah, Solange Kakou-Ngazoa, Sébastien Dutertre

**Affiliations:** 1 Bioinformatic Team, Département Agriculture et Ressource Animales, UMRI 28, Institut National Polytechnique Félix Houphouët-Boigny, Yamoussoukro BP 1093, Ivory Coast; anicet.ebou@gmail.com; 2 Plateforme de Biologie Moléculaire, Institut Pasteur de Côte d’Ivoire, Abidjan BP 490, Ivory Coast; addablah.audrey@gmail.com (A.A.); solangekakou@pasteur.ci (S.K.-N.); 3 Institut des Biomolécules Max Mousseron, UMR 5247, Université de Montpellier, CNRS, ENSCM, 34095 Montpellier, France

**Keywords:** cone snails, venom, conotoxins, antimicrobial peptides, antibacterial activity, proteotranscriptomic approach

## Abstract

Despite their impressive diversity and already broad therapeutic applications, cone snail venoms have received less attention as a natural source in the investigation of antimicrobial peptides than other venomous animals such as scorpions, spiders, or snakes. Cone snails are among the largest genera (*Conus* sp.) of marine invertebrates, with more than seven hundred species described to date. These predatory mollusks use their sophisticated venom apparatus to capture prey or defend themselves. In-depth studies of these venoms have unraveled many biologically active peptides with pharmacological properties of interest in the field of pain management, the treatment of epilepsy, neurodegenerative diseases, and cardiac ischemia. Considering sequencing efficiency and affordability, cone snail venom gland transcriptome analyses could allow the discovery of new, promising antimicrobial peptides. We first present here the need for novel compounds like antimicrobial peptides as a viable alternative to conventional antibiotics. Secondly, we review the current knowledge on cone snails as a source of antimicrobial peptides. Then, we present the current state of the art in analytical methods applied to crude or milked venom followed by how antibacterial activity assay can be implemented for fostering cone snail antimicrobial peptides studies. We also propose a new innovative profile Hidden Markov model-based approach to annotate full venom gland transcriptomes and speed up the discovery of potentially active peptides from cone snails.

## 1. Introduction

In 1928, Alexander Fleming discovered the now widely known Penicillin from a *Penicillium* mold, later identified as *Penicillium rubens* [1]. Sixteen years later, Selman Waksman isolated Streptomycin from *Streptomyces griseus* [2], and together with Penicillin, both these discoveries undoubtedly revolutionized modern medicine. Indeed, such antibiotics were produced in large quantities during the Second World War to successfully treat infectious diseases [3]. Since then, antibiotics have become an integral part of the lives of millions of people as a primary medication for multiple microbial infections. Unfortunately, the misuse and overuse of antibiotics led to the emergence of antimicrobial resistance, which has become one of the major threats to global human health, as pointed out by the World Health Organization [4]. In 2016, 700,000 deaths were reported to be directly imputable to resistant infections [5]. In the same way, a study on global antimicrobial resistance has estimated that by 2050, over 10 million deaths each year and a cumulative USD 100 trillion of economic losses will be directly imputable to drug resistance [6].

At the beginning of the 21st century, the pharmaceutical industry appeared to reach a critical juncture caused by the low number of novel drugs entering the clinical phases [7]. Nevertheless, in the same couple of years, the advent of new sequencing technologies, higher computational capacities, and advances in biological research led the industry and researchers to turn more deeply towards natural extracts like animal venoms as a source of novel drug leads [8]. Back in the 17th century, Francesco Redi led some studies on toxins, especially from the “poisonous” snakes. Some years later, the discovery of snake venom glands set the stage for the birth of modern toxicology [9]. Since then, the iconic scorpions, spiders, and snakes, but also more recently lesser-known invertebrates like cone snails, have been investigated for their venom toxins.

*Conus* sp. is a major group of marine invertebrates, with about 761 described species according to the world register of marine species (WoRMS) [10]. The major component of the venom gland of cone snails is made of small peptides known as conotoxins, which often have neurotoxic activities. It has been conservatively estimated that more than 80,000 biologically active conotoxins are yet to be discovered [11]. The studies of conotoxins have already led to the discovery of the well-known drug ziconotide (ω-MVIIA conotoxin) from *Conus magus* used to treat chronic pain [12,13]. Each species of cone snail produces hundreds of conotoxins, which are the result of years of evolution and coevolution in the same environment as their prey and predators [14]. Venom compounds can be classified based on their molecular targets and mode of action, like peptides with antimicrobial activity. Antimicrobial peptides (AMPs) are biomolecules that act on bacteria, viruses, or fungi. They are usually short polypeptides with a net positive charge that comes from their rich composition in lysine, arginine, and histidine [15].

Compared to other animals like scorpions or spiders, the antimicrobial peptides from cone snails have received little attention [16,17,18]. For example, the investigation of scorpion venoms led to the discovery of Hadrurin, Scorpine, Vejovine, StCT2, Pandinin 1, and Pandinin 2, which are effective antimicrobial peptides [19,20,21,22,23]. Similar studies also found antimicrobial peptides in snakes, spiders, bees, ants, centipedes, lugworms, wasps, and cuttlefish [15,24,25,26,27,28,29,30,31,32].

To fill this gap in cone snail venom studies, we show here the need for novel compounds in the battle against antimicrobial resistance, update the knowledge on cone snails as a source of antimicrobial peptides and present the analytical methods to decipher the venom of cone snail for antimicrobial peptides.

## 2. Global Antimicrobial Resistance and the Need for Novel Compounds

The word “antibiotic” was used by Selman Waksman, in 1941, to describe any small molecule made by a microorganism that antagonizes the growth of other microorganisms [33]. An antibiotic interferes with bacteria growth via a specific mode of action, at concentrations that present minimal toxicity and are sufficiently potent to be effective against the infection. Despite a 36% increase in human use of antibiotics from 2000 to 2010, approximately 20% of deaths worldwide remain related to infectious diseases because of antibiotic resistance [34]. This development of resistance is a normal evolutionary process for microorganisms. Nevertheless, it is worsened by the selective pressure exerted by the widespread use of antibacterial drugs, rendering them largely ineffective on bacterial infections.

In response to exposure to antibiotics, susceptible bacteria are killed. However, there is a resistant portion that recolonizes the infection site. Bacteria use two major mechanisms to “resist” antibiotics, which are mutational adaptation and the acquisition of genetic material through horizontal gene transfer. The latter is one of the most important drivers of bacterial evolution. In general, mutations alter antibiotic action via the structural modification of the antibiotic target site, preventing the molecule from reaching its target by decreasing penetration or actively extruding the antimicrobial compound [35]. Resistance to antibiotics can usually be achieved through multiple biochemical pathways. For example, resistant bacteria can produce enzymes like carbapenemase or beta-lactamases, which can degrade or destroy antibiotic molecules. An analysis of 83 studies conducted in Africa showed a prevalence of carbapenemase-producing bacteria isolated in hospitals ranging from 3% to 67.7%, with a mortality rate of up to 67% [36]. Beta-lactamases-producing bacteria and quinolone-resistant bacteria have been detected in humans, but also animals, and the environment [37,38]. Up to 35% of isolates from animals and 93.2% from humans have shown resistance to quinolones [37].

The increase in drug-resistant pathogens is the consequence of various factors. It is the result of biochemical and genetic modifications, as pointed out above, but also the result of the over-reliance on antibiotics. For example, antibiotics are largely used as growth promoters in livestock farming [39]. Resistant bacteria are, then, transmitted to humans through direct contact with animals [40,41,42], exposure to animal manure, and by the consumption of undercooked meat [43]. High rates of prescriptions of antibiotics are another critical factor in the evolution of resistance. In some countries, antibiotics are available without prescriptions. When they are prescribed, most prescriptions are unsuitable. Broad-spectrum antibiotics are favored to treat infections caused by several species of bacteria or those for which diagnosis is time-consuming. Additionally, some physicians are inclined to prescribe multiple antibiotics for the same condition [44]. Patients’ failure to comply with dosages is also a major contributor to the development of resistance. Indeed, patients miss doses, either by mistake or deliberately. Finally, exposure of surviving microorganisms to subtherapeutic concentrations of the drug also increases the chances of developing resistance [45]. Due to increasing resistance to antibiotics, a report has predicted that by 2050 10 million deaths will occur annually due to resistant pathogens. Without control action, by 2050 the cost of antimicrobial resistance will be approximately USD 100 trillion, with a 3.5% decline in global gross domestic product [6]. As the world is heading towards a postantibiotic era, there is an urgent need for novel compounds that are able to meet current needs, such as antimicrobial peptides.

Antimicrobial peptides (AMPs) are active molecules produced by a broad range of organisms ranging from microbes to mammals to ensure their self-defense against microbes [46]. AMPs are multifaceted molecules that kill microbes via a pleiotropic mechanisms of action, such as the destruction of the barrier function of the cellular membranes and the inhibition of macromolecule synthesis making them efficient even against multiresistant bacteria [47,48]. The efficiency of AMPs is due to rapid killing kinetics, reduced toxicity, and reduced microbial resistance [49] as demonstrated by many studies [50,51,52]. AMPs are therefore a promising alternative to conventional antibiotics.

## 3. Cone snails as a Source of Novel Antimicrobial Peptides

### 3.1. Cone Snails Diversity

**Figure 1:**
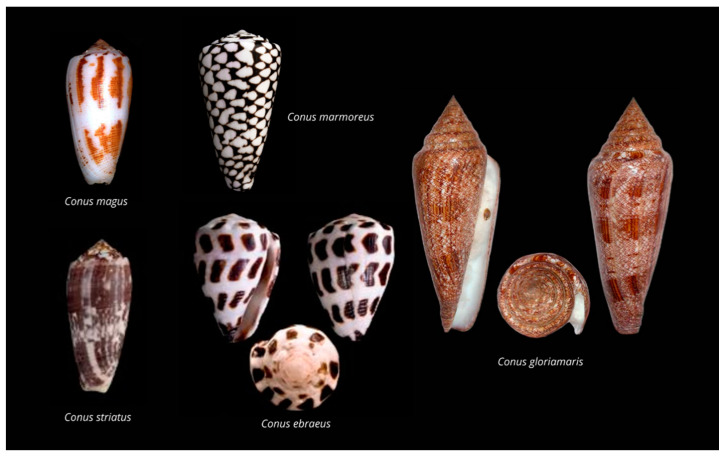
Shells of representative species of *Conus* sp., including fish hunters (*C. magus* and *C. striatus*), mollusk hunters (*C. marmoreus* and *C. gloriamaris*), and a worm hunter (*C. ebraeus*).

The Conidae family is composed of 138 genera from which *Conus* sp. is the type taxon. Cone snails contain around 761 recognized species [53,54] from which approximately 118 have been studied to date (http://conoserver.org/?page=stats&tab=organisms (accessed on 28 January 2021)). However, it should be noted that although this is a rapidly evolving field, especially with the advent of transcriptomics, not all sequences deposited in Conoserver correspond to proteomic-verified conopeptides. The shell patterns of cone snails (Figure 1), clearly recognizable, have made them precious collection items through centuries [55].

Cone snails inhabit tropical and subtropical coastal zones and oceans up to 1000 m of depth but are mostly found in coral reefs hiding in the sand, under the coral shelf, or in shallow waters [56]. The greatest diversity of cone snail species is found in the Indo-Pacific ocean, but they become rarer beyond the 40° N or S parallel [57,58]. Cone snails are active at night as they leave their hiding place to go hunting for prey. They are highly specialized predators and can be classified based on their feeding habits: worm hunters (vermivorous), mollusk hunters (molluscivorous), fish hunters (piscivorous), and generalist feeders. They first detect prey using chemosensors, then crawl softly towards the prey, and generally extend their proboscis and fire their venom-loaded harpoon upon contact. Venom injection into a prey animal induces rapid immobilization, which, in the case of fish hunters, can take less than a few hundred milliseconds [59].

### 3.2. Cone Snail’s Venom Composition and Conotoxins Classification

Conotoxins are the disulfide-rich peptides found in the venom of cone snails responsible for their toxicity [17,60]. They are produced in the venom gland as precursors, which generally contain a signal, a pro, and a mature sequence. The cone snail’s venom is a remarkably complex mixture of peptides that has drawn the attention of biomedical researchers for their high unprecedented potency and selectivity for their target. Conotoxins are classified following three different criteria: the precursor’s endoplasmic reticulum signal sequence, the cysteine pattern of the mature peptide region, and the pharmacological targets. Based on their signal peptide, conotoxins are further divided into 28 gene superfamilies: A, B1, B2, B3, C, D, E, F, G, I1, I2, I3, J, K, L, M, N, O1, O2, O3, P, Q, R, S, T, V, Y. The most expressed peptides from cone snail venom glands studied to date are from A, M, and O superfamilies [61].

Conotoxins can also be divided according to the cysteine pattern or the pharmacological target [62]. Conotoxins have been classified into cysteine frameworks (pattern of cysteine residues) following the arrangement of cysteine along the mature sequence or the disulfide connectivities. The updated cysteine arrangement and disulfide connectivities definition of known conotoxins is presented in Table 1.

Conotoxins exhibit a large range of pharmacological targets. Considering the receptor specificity, we can classify conotoxins into 12 families: α (alpha), γ (gamma), δ (delta), ε (epsilon), ι (iota), κ (kappa), µ (mu), ρ (rho), ς (sigma), τ (tau), χ (chi) and ω (omega) (Table 2). A more detailed description of pharmacological families is provided by Kaas et al. [62].

### 3.3. Antimicrobial Activity of Conidae’s Conopeptides

Although conotoxins have a wide array of unique structures, the most common use of conotoxins is focused on pain management [63]. However, some conotoxins have proved to be highly effective against pathogens with little resistance due to their membrane-disruptive mechanisms [64]. The antimicrobial effects of conotoxins are seldomly documented and remain underexplored. An example of the in vitro antiparasitic activity of conotoxins was demonstrated on the tachyzoite form of *Toxoplasma gondii* [18]. The tachyzoite is the mobile, invasive, and intracellular obligate form of *T. gondii*. The synthetic conotoxin s-cal14.1a derived from *Californiconus californicus*, at micromolar concentration, lowers down to half the viability of extracellular tachyzoites and inhibits host cell invasion by 61%. In toxoplasma infections, no drugs have shown effectiveness when tachyzoites were localized in cytoplasmic parasitophorous vacuoles. However, s-cal14.1a inhibits not only the establishment of the infection but also the intracellular proliferation of *T. gondii* by 50%. This conotoxin potentially disrupts the replication machinery of the parasite by passing the membrane of the host cell, parasitophorous vacuoles, and parasite membranes without the host cell being affected.

Other studies have shown mitigated antibacterial effects of conotoxins. Conotoxin O1_cal29b, isolated from *C. californicus*, inhibited in vitro the growth of the multidrug-resistant *Mycobacterium tuberculosis*. O1_cal29b was effective at low concentration. The inhibition occurred at a minimal inhibitory concentration (MIC) of 0.22–3.52 µM [61]. In parallel, peptide Lo6/7a, a 24-residue conotoxin isolated from the venom of *Conasprella longurionis*, exhibited low and extremely specific activity against *Bacillus megaterium* at an exceedingly high concentration (1 mM) [65]. Additionally, the conolysin-Mt, a disulfide-poor conopeptide from *Conus mustelinus*, showed a low antimicrobial activity with a MIC greater than 50 µM against two *Escherichia coli* strains. Its MIC for the Gram-positive *Staphylococcus aureus* is in a range of 25–50 µM [66].

Interestingly, a study has shown that macrocyclization can convert a conotoxin into an effective antimicrobial peptide [67]. MVIIA is a linear cystine-knot peptide with multiple basic amino acids at both termini, but up to 500 µM, it was inactive against *E. coli*, *Pseudomonas aeruginosa*, and *Staphylococcus aureus*. Meanwhile, it exhibited moderate antifungal activity against *Candida kefyr* and *Candida tropicalis* with MICs of 28.8 µM and 39.8 µM, respectively. Likewise, the linear analog MVIIA-GS, the MVIIA conotoxin with a Gly Ser linker, gave similar results, indicating that the linker did not affect the antimicrobial activity. The authors then considered ligating the two ends of MVIIA using a linker peptide. This ligation allowed the formation of epitopes that gain membranolytic activity on microbes. Indeed, all ten cyclic conotoxins derived from MVIIA were active against the selected three bacterial strains with MICs ranging from 3.3 to 90.2 µM. Most of these analogs exhibited improved antifungal activity with MICs, from up to 18.2 µM against *C. kefyr* and up to 11.4 µM against *C. tropicalis*. Additionally, the conversion of the disulfide bonds to aminobutyric acids improved the antimicrobial activity of the cyclic analogs. These results demonstrated that the end-to-end cyclization of a linear peptide improves its biological activity and confers antimicrobial properties that were not found in the linear form. The mimetics of conopeptides may serve as more promising candidates for the further development of therapeutically useful agents for the treatment of infections.

Although Conolysin-Mt and conotoxin MVIIA target the lipid bilayer of bacteria, in addition to the lipid bilayer, transpeptidase enzymes were explored as novel targets for antimicrobial conopeptides. For example, sortases that anchor surface proteins were investigated as attractive targets due to their prevalence in the cell wall of Gram-positive bacteria [16]. Their inhibition compromises the pathogenesis and the virulence of the bacteria. Based on structural studies, M2-conotoxin and contryphan-R were found to mediate the inhibition of SrtA and SrtB by obstructing the assembly of iron acquisition, immune evasion, complement pathway inhibition, clumps, biofilm formation, and host matrix attachment proteins within the cell wall of *S. aureus*. Therefore, more studies are required to validate the efficacy of M2-conotoxin and contryphan-R in bacterial cultures.

## 4. Bioinformatics-Aided Proteotranscriptomics

### 4.1. Cone Snail Venom Extraction

Cone snail venom can be obtained either by the dissection of the venom duct or by milking. Venom gland tissue extraction implies the availability of tens to hundreds of specimens to collect enough material for the discovery process. Besides the ethical concerns, this method has many disadvantages (cellular debris, unmatured and degraded products in the reconstituted venom) compared to venom “milking” [56]. Although dissection and tissue extraction has proven to be successful in many cases [68], venom milking should be considered whenever possible, as it provides a soluble fraction that contains fully mature conotoxins intended for a particular ecological role, as demonstrated by many studies [69,70,71,72,73,74,75,76]. Nevertheless, if possible, a combination of both strategies (milking and dissection) should be considered as all Conidae venoms tested to date for AMPs have not been milked but rather extracted. Indeed, it was found that cone snails can deploy different combinations of conotoxins depending on the stimulus (predatory vs. defense) [70].

To collect the predation-evoked venom, the procedure usually begins with a lure, which is a live prey according to the cone snail’s feeding habit [77]. The cone extends its proboscis toward the lure that is placed in front of a microtube. The venom is injected into the tube through a fine piece of prey tegument (fish fin for instance). Therefore, milking provides a more soluble venom, free of cellular debris and degraded products, that is ideally suited for biological and proteomic assays [56,78].

### 4.2. Next-Generation Transcriptomics Sequencing and Bioinformatics

**Figure 2:**
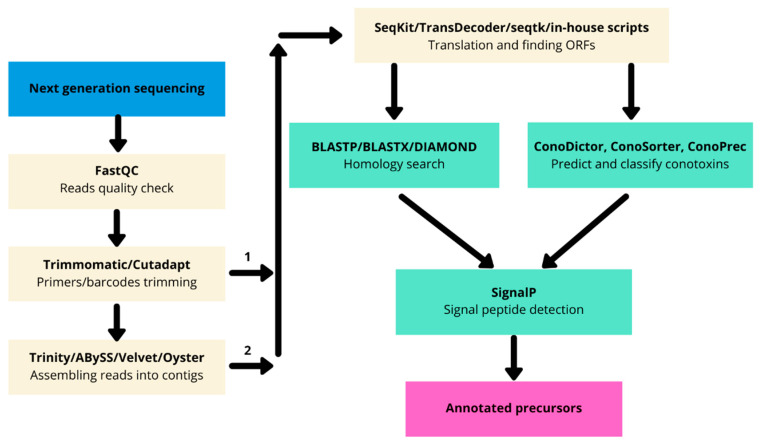
Transcriptomic bioinformatic pipeline for putative conopeptide prediction. Both assembly free (1) and assembly (2) methods are possible. Sequencing: Blue, Preprocessing: Pale brown, Sequence annotation: Pale green, Result: Pink.

Before the advent of next-generation sequencing, biologically active peptides from cone snails were discovered using “bioactivity-guided fractionation”. This method was limited because it required large amounts of crude venom and turned out to be time-consuming [79]. Next-generation sequencing revolutionized the field as the venom duct transcriptome can be completely sequenced and the conotoxin sequences recovered in one single experiment. From the next-generation sequencing data, bioinformatics tools are required to correctly identify conotoxin precursors from the raw reads and/or assembled contigs (Figure 2).

The raw reads obtained from Illumina or 454 sequencings are controlled for quality using FastQC v.0.11.9 (http://www.bioinformatics.babraham.ac.uk/projects/fastqc (accessed on 24 March 2021)). FastQC helps researchers to be aware of some issues that can arise during sequencing by providing statistics on reads like k-mer content, GC content, or graph of quality per base. The validated reads are then trimmed for barcodes and primers using Trimmomatic version 0.39 [80] or Cutadapt version 3.3 [81]. Short Illumina reads obtained after RNA sequencing rarely contain the full-length conotoxin precursor, although some active peptides from cone snails can be as short as 10 amino acids [82]. Therefore, they need to be assembled in longer contigs. De novo assembly can be performed using software like Trinity version 2.12 [83], Velvet version 1.2.10 [84], and ABySS v.2.3.0 [85] to obtain the desired contigs. Another emerging method is the use of multiassembling tools like the Oyster river protocol [86] for a better recovery of contigs, as one method alone is not always able to properly identify all desired contigs.

The contigs are then translated in silico into amino acids. The tools used at this step should be well tested [87]. At this point, the traditional method consists of the homology search using BLAST [88] against a specialized database of conotoxins like ConoServer [89], followed by a second homology search against a larger database like the NCBI nonredundant (https://www.ncbi.nlm.nih.gov/refseq/about/nonredundantproteins accessed on 28 January 2021) [90] or the Uniprot/SwissProt [91] databases for validation of conopeptides and housekeeping genes identification.

### 4.3. Venom Gland Transcriptome Annotation Based on Profile Hidden Markov Models

A second way to elucidate transcriptome composition is by using ConoDictor version 2 [92] (new version in preparation), or ConoSorter [93] to predict putative conotoxins using methods such as hidden Markov models (HMMs). Using specialized predictive tools is helpful because they are exclusively designed to detect putative conopeptides from RNA-seq data and are way speedier compared to the traditional BLAST. Functional and structural annotations like superfamily belonging, signal peptide presence, or cysteine framework can then be predicted using signalP version 5 [94] or previously mentioned tools.

Nevertheless, currently, only a few HMMs are available on PFAM [95] to annotate conotoxins (Table 3).

To accelerate the annotation of cone snail transcriptome, we propose here an up-to-date exhaustive list of profile HMMs (pHMMs) that allow the annotation and classification of the major conotoxins superfamilies (available on our GitHub https://github.com/koualab/cono-amp-review/blob/main/conohmm.txt (accessed on 18 March 2021)). A sequence logo is associated with each of the newly built pHMM (Table 4).

### 4.4. Venom Proteomics

The complex cone snail venom mixture made of polypeptides, peptides, inorganic salt, and amines accounts for most of the molecular and functional diversity as well as the observed behavioral and biochemical modulations [70,76,97]. For the high propensity of post-translational modifications (PTMs) alone, the conopeptides cannot be simply deciphered from transcriptomic data without proteomic evidence. The introduction in the field of mass spectrometry-based techniques has drastically changed the landscape of conotoxin studies [98]. This technique allowed us not only to correctly estimate the number of conopeptides expressed by a cone snail but also improved the resolution of the identified peptides. For instance, LC-MS of milked (injected) venom can help to reveal dramatic intraspecific variation like in some recent studies [97,99,100]. Furthermore, using deep venomics, Dutertre et al., were able to increase the estimation of the number of peptides expressed by *Conus marmoreus* (around 8000 peptides) originated from only 105 conotoxins peptide precursors [69].

### 4.5. Proteotranscriptomics

Proteotranscriptomic studies combine the best of both worlds: de novo deduced sequences of the digested peptides are directly mapped to the RNAseq generated peptide database using dedicated tools. The data obtained through bottom-up proteomics are generally compared to a sequence database using tools like ConoMass [89], MASCOT (Matrix Science, Boston, MA, USA; www.matrixscience.com version 2.5 ProteinPilot (SCIEX, Framingham, MA, USA), v.4.4 or PEAKS studio (Bioinformatics Solutions, Waterloo, ON, Canada) version 8.5. The top-down bioinformatic analyses are more difficult due to the nature of the obtained data. Such data often leads to false-positive identification due to the newness of the sequence variants and PTMs that they represent. To overcome this issue, new and less stringent algorithms have been developed, such as Byonic [101] and ProSight version 2 [102]. Both can be used to identify sequences and PTMs from top-down proteomic data.

### 4.6. In Silico AMPs Structure Determination

Structure determination of AMPs is usually conducted by NMR. However, the potentially large number of sequences identified by transcriptomics, bioinformatics, and proteomics prevent the generalized use of NMR, as it will be too expensive and time-consuming. A better approach involves the in silico determination of the predicted AMPs. With the development of new technologies and algorithms in biophysics, in silico prediction of the structure is becoming more and more precise. Rosetta has proven to be successful in predicting the three-dimensional structure of proteins ab initio from their amino acid sequence. By incorporating new energy functions, Rosetta has been able to predict a completely new protein fold [72,73]. Following a similar strategy, QUARK [103] has shown the highest scores in CASP9 (http://predictioncenter.org/casp9/CD/data/html/groups.server.fm.html (accessed on 28 January 2021)) and CASP10 (http://predictioncenter.org/casp10/groups_analysis.cgi?type=server&tbm=on&tbm_hard=on&tbmfm=on&fm=on&submit=Filter (accessed on 28 January 2021)) challenges. Additionally, the 14th CASP experiment has crowned the i-TASSER web portal (https://zhanglab.ccmb.med.umich.edu/I-TASSER (accessed on 28 January 2021)) as the first protein structure prediction. AlphaFold in CASP13 has shown the greatest advance in the resolution of the protein folding challenges using machine learning methods and, once available, could be used for AMPs structure prediction [104,105].

## 5. Antibacterial Activity Assays

Microbiological bioassays involve the detection and characterization of the antibacterial activity of newly discovered AMPs. Usually, microdilution [66], radial diffusion assay [67], and spot diffusion [65] were used to determine the minimal inhibitory concentration of the tested conopeptide. Quantitative susceptibility testing is performed by making 2-fold dilutions of the tested AMP in a liquid culture medium inoculating it with a standard number of microorganisms (10.5 to 10.6 colony-forming unit (cfu)/mL) and incubating it at 35–37 °C for 18–24 h. Mueller–Hinton Broth is the recommended medium for the susceptibility testing of commonly isolated, rapidly growing aerobic or aeroanaerobic organisms because it supports the satisfactory growth of most pathogens [106]. However, LB medium and Tryptic Soy Broth can also be used [66,67]. The amount of AMP that inhibits the visible growth of the microorganism is called the minimal inhibitory concentration [107]. Beyond MIC, minimal bactericidal concentration (MBC) can be determined to characterize the type of activity performed by the conopeptide. Subcultures of the samples obtained from the clear tubes or wells are plated on a solid medium and reincubated for an additional 18–24 h. The MBC represents the lowest concentration that either revealed no visible bacterial growth after subculturing or resulted in a 3-log10 reduction in colony-forming units (cfu) per mL on subculture. Briefly, a 3-log10 (or 99.9%) reduction in viable bacterial count in an 18–24 h period is the accepted definition of bactericidal activity [108]. The bacteriostatic activity has been defined as a ratio of MBC to MIC > 4 [81].

In case of low activity, different peptide mixtures can be tested in combination to increase the bactericidal activity and the synergistic antimicrobial effect evaluated. The fractional inhibitory concentration (FIC) index is defined as the inhibitory concentration of the antimicrobial combination divided by that of the single antimicrobial component [109]. The following equation (Equation (1)) represents the method for determining the FIC index:

(1) $$FICindex=\frac{MICofdrugX\in combination}{MICofdrugXalone}+\frac{MICofdrugY\in combination}{MICofdrugYalone}$$

The FIC indices are interpreted as follows: ≤0.5 = synergism; 0.5–1 = additivity; 1–4 = indifference; and >4 = antagonism.

Lastly, to distinguish if the antibacterial activity is concentration and/or time-dependent, time-kill curves can be performed. Viable colony counts are determined at different time points up to 24 h. The cfu of the organisms is to be determined and a graph of the log cfu/mL is plotted against time. This kinetics can also be related to the stages of the growth of the bacteria (lag, exponential, stationary phase). Concentration-dependent bactericidal activity occurs when the rate of killed-bacteria increases with progressively higher AMP concentrations corresponding to multiples of the MIC (i.e., 1×, 5×, 10×). Time-dependent bactericidal activity occurs when bacterial killing does not change with increasing AMPs concentrations to more than the MIC.

## 6. Conclusions

Cone snail venom has already been the source of approved toxin-based drugs, and these mixtures are also potential reservoirs for antimicrobial peptides. We first reported the standard methods in venom drug-based research for transcriptome sequencing and proteomics. We further showed a new strategy based on a dedicated predictive approach focused on up-to-date profile Hidden Markov models that should then make it easy to quickly identify potentially interesting putative peptides from cone snail venom gland transcriptomes. We also pointed out the proteomics and proteotranscriptomics methods to confirm conotoxin transcriptome-based prediction. Furthermore, a point on the in silico prediction of conotoxins’ three-dimensional structure has been made. We conclude our review by mentioning the antibacterial activity assay methods for the proper assay of conotoxins AMPs. We hope that this review will help researchers and industry to lead projects on cone snail antimicrobial peptides to tackle the increasing global antimicrobial resistance.

## Figures and Tables

**Table 1:** Definition of conotoxins cysteine frameworks along with cysteine spacing and disulfide connectivity. Adapted from Kaas et al. [62].

Name.	Number of Cysteines	Cysteine Pattern	Disulfide Connectivity
I	4	CC-C-C	I–III, II–IV
II	6	CCC-C-C-C	
III	6	CC-C-C-CC	(I–IV, II–V, III–VI), (I–VI, II–IV, III–V), (I–V, II–IV, III–VI)
IV	6	CC-C-C-C-C	I–V, II–III, IV–VI
V	4	CC-CC	I–III, II–IV
VI/VII	6	C-C-CC-C-C	I–IV, II–V, III–VI
VIII	10	C-C-C-C-C-C-C-C-C-C	
IX	6	C-C-C-C-C-C	I–IV, II–V, III–VI
X	4	CC-C-C	I–IV, II–III
XI	8	C-C-CC-CC-C-C	I–IV, II–VI, III–VII, V–VIII
XII	8	C-C-C-C-CC-C-C	
XIII	8	C-C-C-CC-C-C-C	
XIV	4	C-C-C-C	I–III, II–IV
XV	8	C-C-CC-C-C-C-C	
XVI	4	C-C-CC	
XVII	8	C-C-CC-C-CC-C	
XVIII	6	C-C-CC-CC	
XIX	10	C-C-C-CCC-C-C-C-C	
XX	10	C-CC-C-CC-C-C-C-C	
XXI	10	CC-C-C-C-CC-C-C-C	
XXII	8	C-C-C-C-C-C-C-C	
XXIII	6	C-C-C-CC-C	
XXIV	4	C-CC-C	
XXV	6	C-C-C-C-CC	
XXVI	8	C-C-C-C-CC-CC	
XXVII	8	C-C-C-CCC-C-C	
XXVIII	10	C-C-C-CC-C-C-C-C-C	
XXIX	8	CCC-C-CC-C-C	
XXX	10	C-C-CCC-C-C-C-CC	
XXXII	6	C-CC-C-C-C	
XXXIII	12	C-C-C-C-C-C-C-C-C-C-C-C	

**Table 2:** Pharmacological families of conotoxins. The UniProt version 2021_1 (https://uniprot.org) accession number of the representative conotoxins is indicated in the parenthesis. Adapted from Kaas et al. [62].

Pharmacological Family	Definition	Conotoxin Representative
α (alpha)	Nicotinic acetylcholine receptors	Alpha-conotoxin GIA (P01519)
γ (gamma)	Neuronal pacemaker cation currents (inward cation current)	Gamma-conotoxin PnVIIA (P56711)
δ (delta)	Voltage-gated Na channels (agonist, delay inactivation)	Delta-conotoxin TxVIA (Q9U655)
ε (epsilon)	Presynaptic Ca channels or G protein-coupled presynaptic receptors	Epsilon-conotoxin TxVA (P81755)
ι (iota)	Voltage-gated Na channels (agonist, no delayed inactivation)	Iota-conotoxin RXIA (Q7Z094)
κ (kappa)	Voltage-gated K channels (blocker)	Kappa-conotoxin PVIIA (P56633)
µ (mu)	Voltage-gated Na channels (antagonist, blocker)	Mu-conotoxin GIIIA (P01523)
ρ (rho)	Alpha1-adrenoceptors (GPCR)	Rho-conotoxin TIA (P58811)
ς (sigma)	Serotonin-gated ion channels 5-HT3	Sigma-conotoxin GVIIIA (P58924)
τ (tau)	Somatostatin receptor	Tau-conotoxin CnVA (P0DJL6)
χ (chi)	Neuronal noradrenaline transporter	Chi-conotoxin MrIA (P58808)
ω (omega)	Voltage-gated Ca channels (blocker)	Omega-conotoxin GVIA (P01522)

**Table 3:** Current conotoxins HMMs profiles available in PFAM.

Accession	ID	Description
PF16981	Chi-conotoxin	chi-Conotoxin or t superfamily
PF02950	Conotoxin	Conotoxin
PF17557	Conotoxin_I2	I2-superfamily conotoxins
PF05374	Mu-conotoxin	Mu-Conotoxin
PF07473	Toxin_11	Spasmodic peptide gm9a; conotoxin from *Conus* species
PF07829	Toxin_14	Alpha-A conotoxin PIVA-like protein
PF08087	Toxin_18	Conotoxin O-superfamily
PF08088	Toxin_19	Conotoxin I-superfamily
PF08094	Toxin_24	Conotoxin TVIIA/GS family
PF08097	Toxin_26	Conotoxin T-superfamily
PF07365	Toxin_8	Alpha conotoxin precursor

**Table 4:** Updated profile HMM (pHMM) of major conotoxins superfamily sequence signal and their associated sequence logo [96].

Conotoxin Superfamily	pHMM	Cysteine Framework	Sequence Logo
A	CN_A	I, II, IV, VI/VII, XIV, XXII	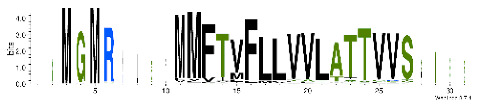
B	CN_B	(conantokins, disulfide-poor)	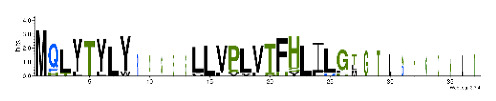
D	CN_D	XXVIII, IV, XIV, XV, XX, XXIV	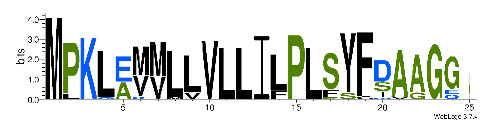
H	CN_H	VI/VII	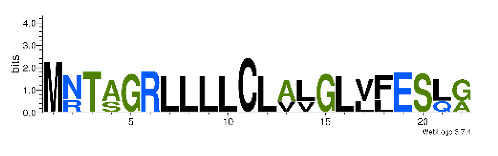
I1	CN_I1	VI/VII, XI, XXII	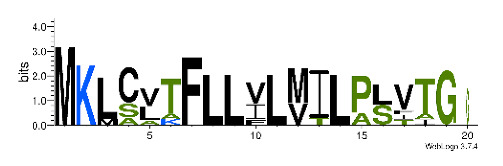
I2	CN_I2	VI/VII, XI, XII, XIII, XIV	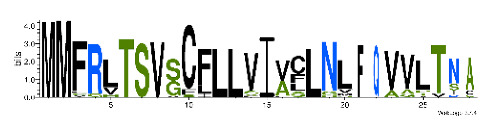
I3	CN_I3	VI/VII, XI	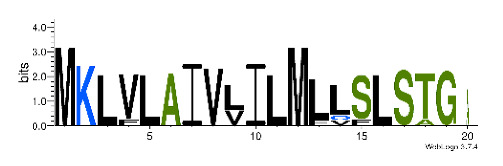
J	CN_J	XIV	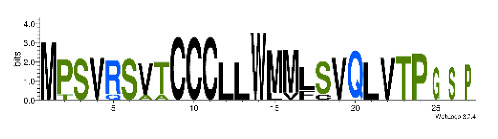
K	CN_K	XXIII	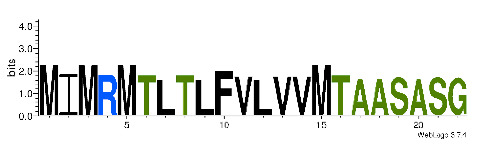
L	CN_L	XIV, XXIV	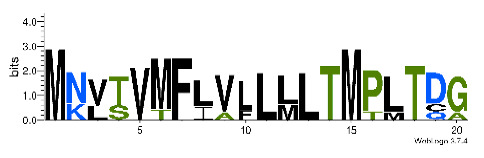
M	CN_M	XXXII, I, II, III, IV, VI/VII, IX, XIV, XVI	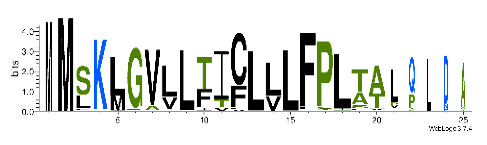
N	CN_N	XV	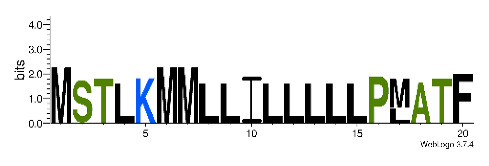
O1	CN_O1	XXIX, I, VI/VII, IX, XII, XIV, XVI	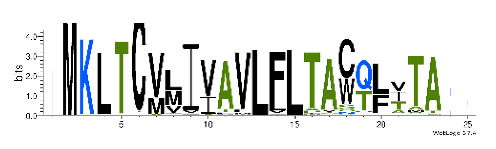
O2	CN_O2	VI/VII, XIV, XV, XVI	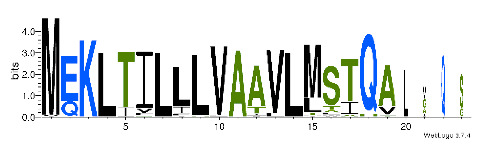
O3	CN_O3	VI/VII, XVI	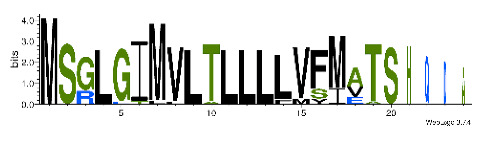
P	CN_P	IX, XIV	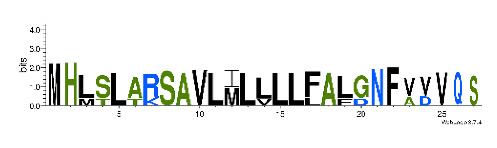
R	CN_R	XIV	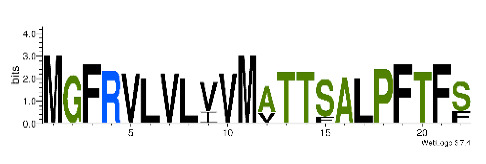
S	CN_S	XXXIII, VIII	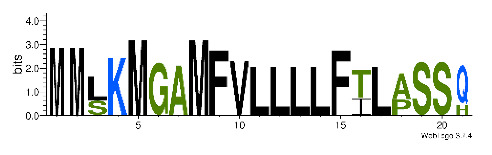
T	CN_T	I, V, X, XVI	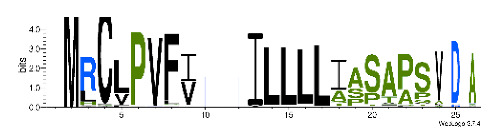

## Data Availability

Not applicable.
